# The Identification of Novel Protein-Protein Interactions in Liver that Affect Glucagon Receptor Activity

**DOI:** 10.1371/journal.pone.0129226

**Published:** 2015-06-15

**Authors:** Junfeng Han, Ming Zhang, Sean Froese, Feihan F. Dai, Mélanie Robitaille, Alpana Bhattacharjee, Xinyi Huang, Weiping Jia, Stéphane Angers, Michael B. Wheeler, Li Wei

**Affiliations:** 1 Department of Physiology and Medicine, University of Toronto, Toronto, Ontario, Canada; 2 Department of Endocrinology and Metabolism, Shanghai Jiao Tong University Affiliated Sixth People’s Hospital, Shanghai, China; 3 Leslie Dan Faculty of Pharmacy, University of Toronto, Toronto, Ontario, M5S1A8, Canada; Van Andel Research Institute, UNITED STATES

## Abstract

Glucagon regulates glucose homeostasis by controlling glycogenolysis and gluconeogenesis in the liver. Exaggerated and dysregulated glucagon secretion can exacerbate hyperglycemia contributing to type 2 diabetes (T2D). Thus, it is important to understand how glucagon receptor (GCGR) activity and signaling is controlled in hepatocytes. To better understand this, we sought to identify proteins that interact with the GCGR to affect ligand-dependent receptor activation. A Flag-tagged human GCGR was recombinantly expressed in Chinese hamster ovary (CHO) cells, and GCGR complexes were isolated by affinity purification (AP). Complexes were then analyzed by mass spectrometry (MS), and protein-GCGR interactions were validated by co-immunoprecipitation (Co-IP) and Western blot. This was followed by studies in primary hepatocytes to assess the effects of each interactor on glucagon-dependent glucose production and intracellular cAMP accumulation, and then in immortalized CHO and liver cell lines to further examine cell signaling. Thirty-three unique interactors were identified from the AP-MS screening of GCGR expressing CHO cells in both glucagon liganded and unliganded states. These studies revealed a particularly robust interaction between GCGR and 5 proteins, further validated by Co-IP, Western blot and qPCR. Overexpression of selected interactors in mouse hepatocytes indicated that two interactors, LDLR and TMED2, significantly enhanced glucagon-stimulated glucose production, while YWHAB inhibited glucose production. This was mirrored with glucagon-stimulated cAMP production, with LDLR and TMED2 enhancing and YWHAB inhibiting cAMP accumulation. To further link these interactors to glucose production, key gluconeogenic genes were assessed. Both LDLR and TMED2 stimulated while YWHAB inhibited PEPCK and G6Pase gene expression. In the present study, we have probed the GCGR interactome and found three novel GCGR interactors that control glucagon-stimulated glucose production by modulating cAMP accumulation and genes that control gluconeogenesis. These interactors may be useful targets to control glucose homeostasis in T2D.

## Introduction

Glucagon, released from pancreatic islet alpha cells, promotes glycogenolysis and gluconeogenesis in the liver to elevate blood glucose levels during fasting. This effect is mediated via its cognate receptor, GCGR. As a member of the class B G protein coupled receptor (GPCR) family, GCGR acts primarily through Gs (PKA-cAMP pathway) but also through Gq, involving phospholipase C (PLC) [1]. Upon receptor activation by glucagon, Gs alpha is released to activate adenylate cyclase and increase intracellular cAMP levels, subsequently activating protein kinase A (PKA) [2]. In addition, the stimulation of Gq leads to the activation of PLC, and the subsequent release of intracellular calcium [3, 4]. As a hormone released in response to hypoglycemia, glucagon is critical in maintaining glucose homeostasis. Elevated glucagon secretion and GCGR activity was observed in diabetes patients [5]. Additionally, disruption of glucagon activity was shown to improve hyperglycemia in ob/ob mice [6]. Therefore antagonists towards the GCGR are considered to be a potential strategy to treat diabetes leading to the development of a number of GCGR antagonists [7, 8]. The first GCGR antagonist identified was the small molecule skyrin, a fungal bisanthroquinone, which was found to inhibit glucagon-stimulated cAMP formation and glucose output from rat and human hepatocytes [9].Later, another GCGR antagonist, Cpd-A, was shown in preclinical models to lower blood glucose, but circulating glucagon and glucagon-like peptide 1 (GLP-1) levels were moderately elevated [10]. Thus, the movement of these compounds to clinical trials was limited by their relatively poor potency/specificity. To facilitate the discovery of novel GCGR antagonists there is a need for a comprehensive understanding of factors/proteins involved in the regulation of its activity and cell signaling.

Over the past decade, GPCR accessory proteins have received significant attention in an effort to explain the diverse functions of the receptors, such as KCTDs to the GABA_B_ receptor [11] and beta-arrestin 1 to the GLP-1 receptor [12]. Although the discovery of novel accessory proteins for other GPCRs is unfolding, the interactome (interacting protein network) of GCGR has yet to be reported. Daulat et al. were the first group to apply an affinity purification and mass spectrometry (AP-MS) approach to reveal the interactome of a GPCR: melatonin receptor 1 and 2 [13]. In addition, several studies have employed AP-MS to identify interactors of both cytosolic and membrane bound proteins [14–16]. We recently identified a set of novel GLP-1R interactors in CHO and MIN6 β cells expressing GLP-1R using a similar AP-MS method which revealed 99 potential interactors [17]. Following validation, one of these novel GLP-1R interactors, PGRMC1, was shown to significantly enhance GLP-1 stimulated insulin secretion [17]. In the present study, we have employed an AP-MS screening approach to identify a GCGR interactome. This method allowed us to study the GCGR in both its unliganded (quiescent) and liganded (activated) states, in mammalian cells.

## Methods

### Animals and cell culture

Mice of C57BL/6 background were used for experiments at ages of 8–12 weeks. Experiments were approved by the Animal Care Committee (University of Toronto) and animals were handled according to the Canadian Council of Animal Care guidelines. Stable human GCGR expressing human liver carcinoma (HepG2-GCGR) cells generated for this study were cultured with high glucose Dulbecco’s Modified Eagle Medium (DMEM) supplemented with 10% fetal bovine serum and 1% penicillin-streptomycin, as were Chinese hamster ovary (CHO) cells. Cells were passaged every 3–5 days. Transient transfections in HepG2-GCGR and CHO cells were performed using Lipofectamine 2000 following the manufacturer’s protocol (Invitrogen, Carlsbad, California).

### Isolated mouse hepatocytes

Mice (C57BL/6) aged 8–12 weeks were fasted overnight and primary hepatocytes were isolated and cultured as previously described [18]. Briefly, primary hepatocytes were isolated using collagenase IV (Sigma, Canada) perfusion. Cells were seeded using DMEM supplemented with 1 g/L glucose, 10 M/L sodium lactate, 0.01 μM/L dexamethasone, 5 mM/L HEPES, and 2 mM/L L-Glutamine. Transient transfections were performed using Lipofectamine 2000 following the manufacturer’s protocol.

### Affinity precipitation and LC-MS/MS (AP-MS)

**Fig 1:**
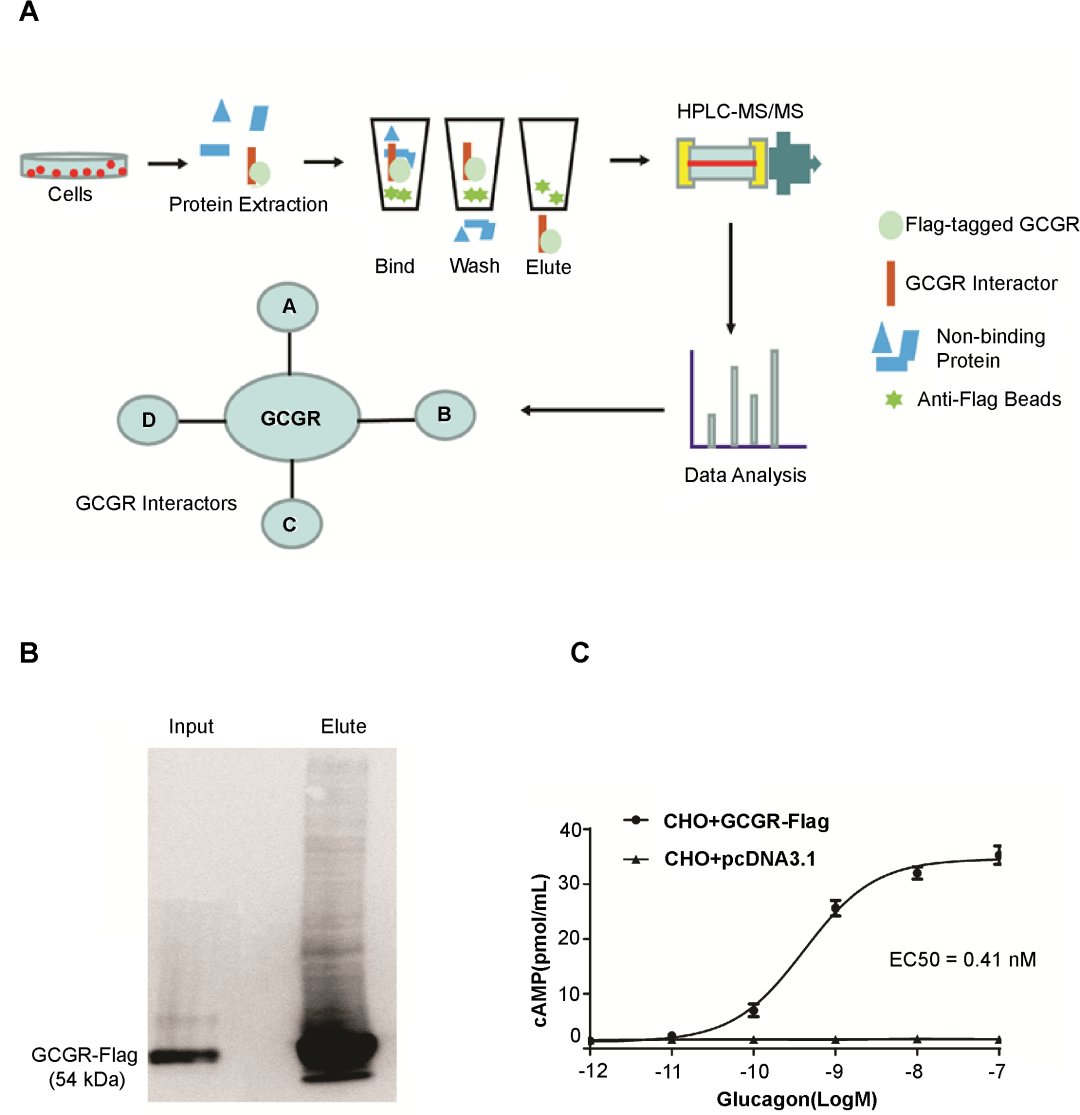
Study design and protocol. A) Flow-chart of the AP-MS method for discovering novel GCGR interactors. CHO cells transfected with pcDNA3.1 were used as a negative control. CHO cells transfected with GCGR-Flag were stimulated with 10 nM glucagon for 5 minutes. Each condition was repeated three times. After protein extraction, anti-Flag Co-IP was conducted using M2 anti-Flag affinity gel, and affinity beads were then washed with wash buffer and ammonium bicarbonate three times before elution by hydroxide (pH 11.0). Co-IP elutes were then used for in-solution trypsin digestion and HPLC-MS/MS (LTQ-XL) to identify GCGR interacting proteins. B) GCGR-Flag is expressed in CHO cells transfected with human GCGR-Flag plasmid. Immunoprecipitation was conducted using M2 anti-Flag affinity gel, 5% lysates (Input, I) and Co-IP elutes (E) loaded on 10% SDS-PAGE gel for electrophoresis. Flag M2 monoclonal antibody was used for Western blots. C) GCGR-Flag transfected CHO cells respond to glucagon stimulation through changes in cAMP levels. EC_50_ = 0.41 nM. GCGR-Flag transfected CHO cells were used for glucagon stimulation (0.001 nM, 0.01 nM, 0.1 nM, 1 nM, 10 nM, 100 nM) for 30 minutes for the cAMP ELISA assay.

Fig 1A schematically outlines the approach used to screen for potential GCGR interacting proteins. Proteins from non-transfected (NT) CHO cells and GCGR-Flag transfected CHO cells with or without glucagon treatment (10 nM) were collected using lysis buffer (10% glycerol, 50 mM/L HEPES (pH 7.4), 150 mM/L NaCl, 2 mM/L EDTA, 0.25% DDM (n-Dodecyl-β-D-maltoside) supplemented with protease inhibitor cocktail (Roche,Basel, Switzerland). Anti-Flag M2 affinity gel (Sigma-Aldrich, Canada) was used to precipitate the GCGR/interactor complex. Anti-Flag M2 beads were washed three times with lysis buffer followed by three washes with 50 mM/L ammonium bicarbonate (pH 8.0). Protein complexes were eluted by 500 mM/L ammonium hydroxide (pH 11.0), and then lyophilized for digestion using sequence grade trypsin (Promega, PR-V5111).

The tryptic mixture was then injected for LC-MS/MS analysis (LTQ-XL) according to the procedure reported previously [19]. Briefly, reverse phase (RP) analytical columns were made of 14–19 cm of RP material (Jupiter 4 mm Proteo 90A; Phenomenex, Inc.). Peptides were eluted by a 2 hour gradient method in which aqueous buffer A is progressively mixed with higher proportions of organic buffer B (5% water, 95% acetonitrile and 0.1% formic acid) in RP columns by the HPLC with a flow-rate of 20–50 nL/min. Peptide ions were dynamically selected for fragmentation using data-dependent acquisition by the operating software (the five most intense precursor ions of each MS scan were selected for subsequent tandem mass spectrometry [MS/MS]). The resulting peptide identifications returned by SEQUEST were filtered and assembled into protein identifications using the transproteomic pipeline software running on a Sorcerer platform (SageNResearch). Mascot was searched with a fragment ion mass tolerance of 0.6 Da and a parent ion tolerance of 3.0 Da. Mascot (matrix Science) version 2.3.02 was used for mouse or human database searching. We used the Prohits software (http://www.prohitsms.com) to analyze the MS data and generate a comparative table regarding protein identification information. We filtered the results using the following criteria/parameters: 1) “mascot score > 50”, 2) “not nuclear, mitochondrial, cytoskeleton and heat shock proteins”, 3) “identified at least 2 times in 3 runs”.

### Plasmids and regents

cDNA of human GCGR (c-terminal Flag-tagged) and GCGR interactors (c-terminal HA-tagged) were constructed in pcDNA3.1. The purified plasmids were prepared using the Midi-Prep kit (Qiagen, Toronto, Canada).

### Western blot

For phosphorylation experiments, HepG2-GCGR cells were transfected with YWHAB and allowed to recover for 48 hours. HepG2-GCGR cells were then pre-incubated in DMEM supplemented with 0.5% fatty acid free BSA for 30 minutes. HepG2-GCGR cells were then stimulated with 100 nM/L glucagon in DMEM supplemented with 0.5% fatty acid free BSA for 15 minutes. Total cell lysate was collected using the same lysis buffer used in affinity purification with the addition of phosphatase inhibitors (10 mM/L sodium orthovanadate and sodium fluoride). Anti-phosphoserine antibody (1:1000 dilution; Sigma-Aldrich, United States) and HRP-conjugated mouse secondary antibody were used for detection of total serine phosphorylation. The membranes were developed with the ECL advance kit (GE Healthcare) and imaged using the Kodak ImageStation4000 Pro (Care stream Health Inc, Rochester, New York).

### Glucose production assay

Primary hepatocytes (2×10^5^ cells per well in twelve-well plates) were first serum starved overnight prior to stimulation. Following serum starving, primary hepatocytes were preincubated with glucose-free DMEM without phenol red for 2 hours. Next, cells were washed with PBS and stimulated with forskolin (10 μM/L), an adenylate cyclase activator, or glucagon (100 nM/L) in glucose-free DMEM without phenol red for 4 h. The culture media were collected for measuring glucose concentration using the Glucose (GO) assay kit (Sigma, Canada). The readings were then normalized to total protein content using the Bradford assay.

### cAMP assay

Intracellular cAMP content was measured in primary hepatocytes as described previously [20]. Briefly, cells were washed with cold PBS and harvested using 80% ethanol. The cell lysates were centrifuged and the supernatant was collected and lyophilized using a SpeedVac. The pellet was resuspended in cAMP assay buffer (0.05 mM/L sodium acetate (pH 6.2) and 0.01% sodium azide) and measured using an intracellular cAMP ELISA kit (Biomedical Technologies Inc, US) in primary hepatocytes. In CHO and HepG2-GCGR cells, cAMP content was measured using the Cisbio cAMP cell-based assay kit according to the manufacturer’s instructions [21].

### Quantitative real-time PCR

Total RNA from primary hepatocytes was extracted using an RNA-easy kit (Qiagen, Canada). cDNA generated by SuperScript II enzyme(Invitrogen, Canada) was analyzed by qPCR using Power SYBR Green PCR master mix following the manufacturer’s protocol (Applied Biosystems, Carlsbad, California) and Vii 7 Real-Time PCR System (Life-Technology, Canada). All data was normalized to β-actin expression. Primers for PCR were designed using the Primer3 software program. Sequences for primers used for this study are provided in S1 Table. Relative gene expression was estimated by the standard curve method [22].

### GCGR binding assay

CHO cells seeded in 6-well plates were transiently co-transfected with Flag-tagged GCGR and HA-tagged YWHAB or pcDNA3.1 and allowed to recover for 48 hours. Cells were then washed twice in PBS and harvested using 2 mM/L EDTA in PBS. Cells (5x10^5^/tube) were incubated at 37°C in binding buffer (DMEM, 1% BSA, pH 7.4) with I^125^ labeled glucagon using a range from 10^−12^ to 10^−6^ M/L in a final volume of 200 μl. Cell suspensions were then centrifuged at 12,000 x g and radioactivity was counted using the Packard Cobra II Gamma Counter (GMI, Ramsey, Minnesota, USA) [23].

### Statistics and bioinformatics

The data are presented as the mean ± SE. Student’s t-test was used to measure the mean difference for measurements of glucose production and cAMP in primary hepatocytes. One-way ANOVA was used to measure the mean difference for cAMP production in CHO and HepG2-GCGR cells. Differences were considered statistically significant at p< 0.05. Cytoscape (http://www.cytoscape.org) was used to generate a GCGR interactor network schematic [24]. We also conducted functional enrichment analysis for GCGR interactors using David Gene Ontology tool (http://david.abcc.ncifcrf.gov). The p value (EASE Score, a modified Fisher Exact p-Value) less than 0.05 means the input interactors’ list is specifically associated (enriched) in certain biological processes.

## Results

### Identification of GCGR interacting proteins using AP-MS

To study the GCGR interactome under liganded and unliganded states, CHO cells expressing GCGR were treated with or without glucagon, followed by AP-MS analyses (**Fig 1A**). By Western blot, we showed that the Flag-tagged GCGR was detected in total lysate and following affinity purification using anti-Flag affinity gel (**Fig 1B**). Importantly, intracellular cAMP accumulation was increased in response to incremental glucagon in GCGR overexpressing CHO cells with the EC_50_ of 0.41 nM (**Fig 1C**), which is consistent with previous reports [25] indicating the GCGR signaling pathway was properly integrated.

**Fig 2:**
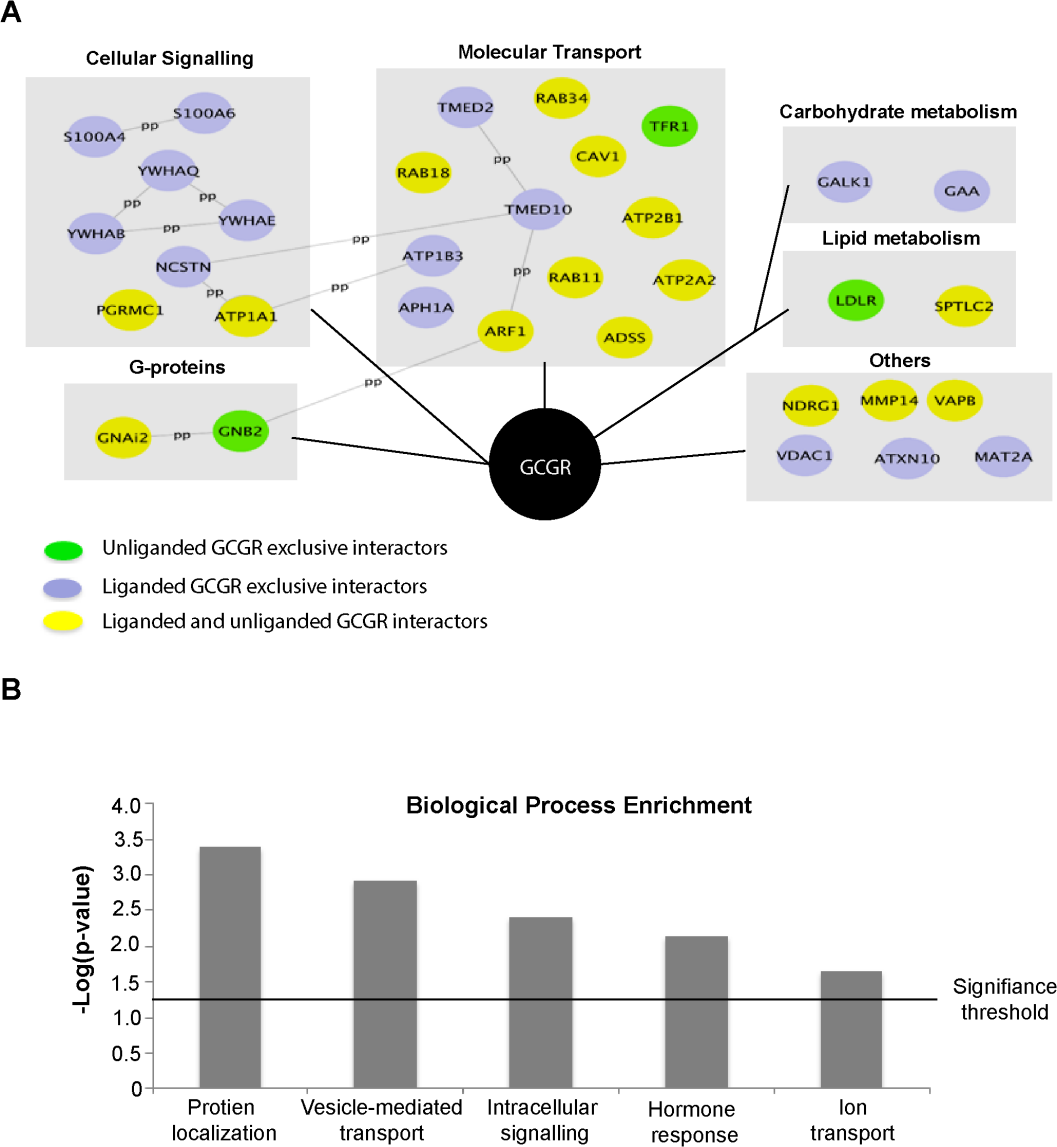
A) The network of GCGR interactors and their functional clusters revealed by AP-MS. Biological functions were retrieved from the Uniprot database. Each gray line indicates known protein-protein interactions extracted from the String database. Green nodes denote those interactors only identified under unliganded state. Blue nodes represent exclusively liganded interactors. Yellow nodes are interactors that were found in both liganded and unliganded states. B) Biological process enrichment analysis showed GCGR interactors are enriched with respect to specific biological functions, as indicated by the DAVID bioinformatics tool. Shown here are biological processes that have an EASE Score <0.05 (a modified Fisher Exact p-Value).

After AP-MS was performed in triplicate, 33 interactors were identified in both liganded and unliganded states (**Fig 2A**), according to the criteria of 1) not identified in control group, 2) not nucleus, ribosome, cytoskeleton or heat shock proteins, 3) identified with at least one unique peptide by MS/MS, and 4) identified at least twice. A complete list of the identified GCGR interactors and their function can be found in S2 Table. Among these 33 interactors, 3 proteins (LDLR, GNB2 and TFR1) were only identified in the unliganded GCGR condition, while 15 proteins (ATP1B3, VDAC1, ATNC10, S100A4, GAA, APH1A, YWHAB, S100A6, NCSTN, YWHAE, TMED10, YWHAQ, MAT2A, GALK1, TMED2) were only found, as tested, to bind to liganded GCGR. The remaining 15 proteins (RAB18, RAB11, ATP2B1, CAV1, ADSS, SPTLC2, PGRMC1, ARF1, MMP14, NDRG1, ATP1A1, ATP2A2, RAB34, GNAi2, VAPB) were identified in both the unliganded and liganded states (**Fig 2A**). By using the Uniprot protein database, we found that these putative interactors were largely associated with three functional clusters; cellular signaling, molecular transport, and carbohydrate/lipid metabolism (**Fig 2A**).

Among 33 putative interacting proteins, only Gs alpha (i) was shown to be related to GCGR function previously [1], while the remaining proteins have never been reported to interact with GCGR. Interestingly, some of the proteins such as CAV1 and PGRMC1 were previously found to interact with GLP-1R [17, 26]. We also performed functional enrichment analysis using the David bioinformatics tool. Examining Gene Ontology biological processes, the 33 GCGR interacting proteins were implicated functionally in protein localization (p = 0.0004), vesicle mediated transport (p = 0.0012), intracellular signaling cascade (p = 0.0039), response to hormone stimulus (p = 0.0074) and ion transport (p = 0.02) (**Fig 2B**).

### Validation of the Interaction between GCGR and Identified Interactors

**Fig 3:**
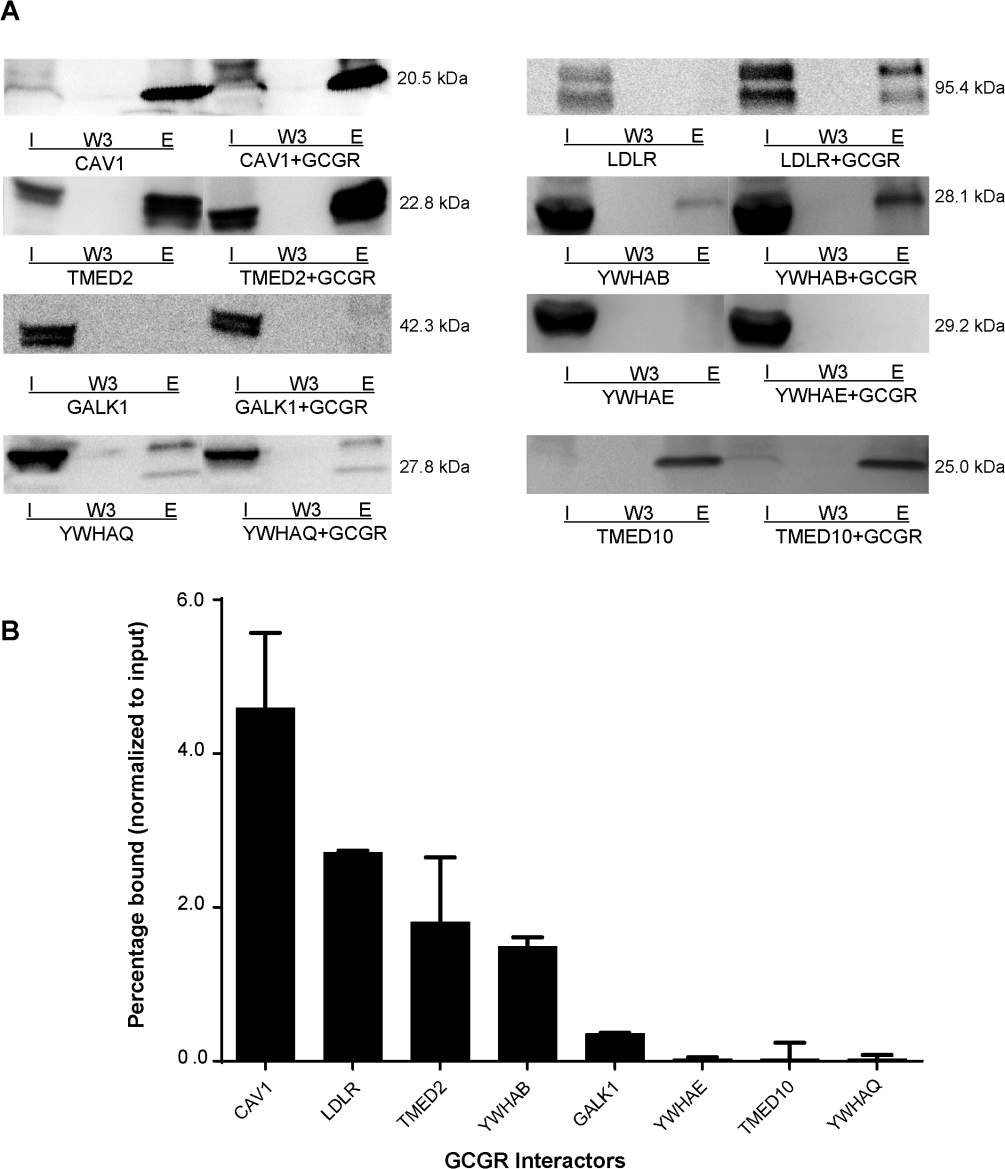
Validation of novel GCGR interactors by Co-IP/WB in CHO cells. HA-tagged interactors and Flag-tagged GCGR were co-transfected into CHO cells, while cells only transfected with HA-tagged interactors were used as a control. Anti-Flag co-immunoprecipitation (Co-IP) and anti-HA Western blot were performed. A) Representative gels from the Co-IP/WB. I = Input proteins before Co-IP, W3 = 3^rd^ wash, E = co-IP elutes. N = 3 per group. B) The relative binding strength for each interactor. For each of the interacting proteins, intensity of the elute/input band was quantified and expressed as a ratio to the lysate band (E:I ratio). The E:I ratio was used to estimate the percentage of input interactors binding to GCGR.

Based on the criteria of 1) membrane bound or cytosol proteins and 2) functional relevance to receptors, we selected 8 interactors (LDLR, CAV1, YWHAQ, YWHAB, YWHAE, TMED2 and TMED10, GALK1) and validated their interaction with GCGR by Co-IP and Western blot (**Fig 3A**).For each interacting protein, intensity of the elute/input band was quantified and expressed as an E:I ratio. The percentage of specific binding was calculated by subtracting the unspecific binding intensities from the control group [17]. The E:I ratio was used to estimate the binding strength/percentage of interactors to GCGR (**Fig 3B**). Among the 8 proteins examined, 5 (CAV1, LDLR, TMED2, YWHAB, GALK1) were prominently detected in anti-Flag Co-IP elutes, suggesting their strong interaction with GCGR (**Fig 3A and 3B**). YWHAE was not detected in the Co-IP elutes, and TMED10 and YWHAQ showed strong binding to anti-Flag affinity gel. These later 3 proteins did not pass the validation test and therefore were not selected for functional studies.

### Selected GCGR interactors affect glucose production in primary mouse hepatocytes

**Fig 4:**
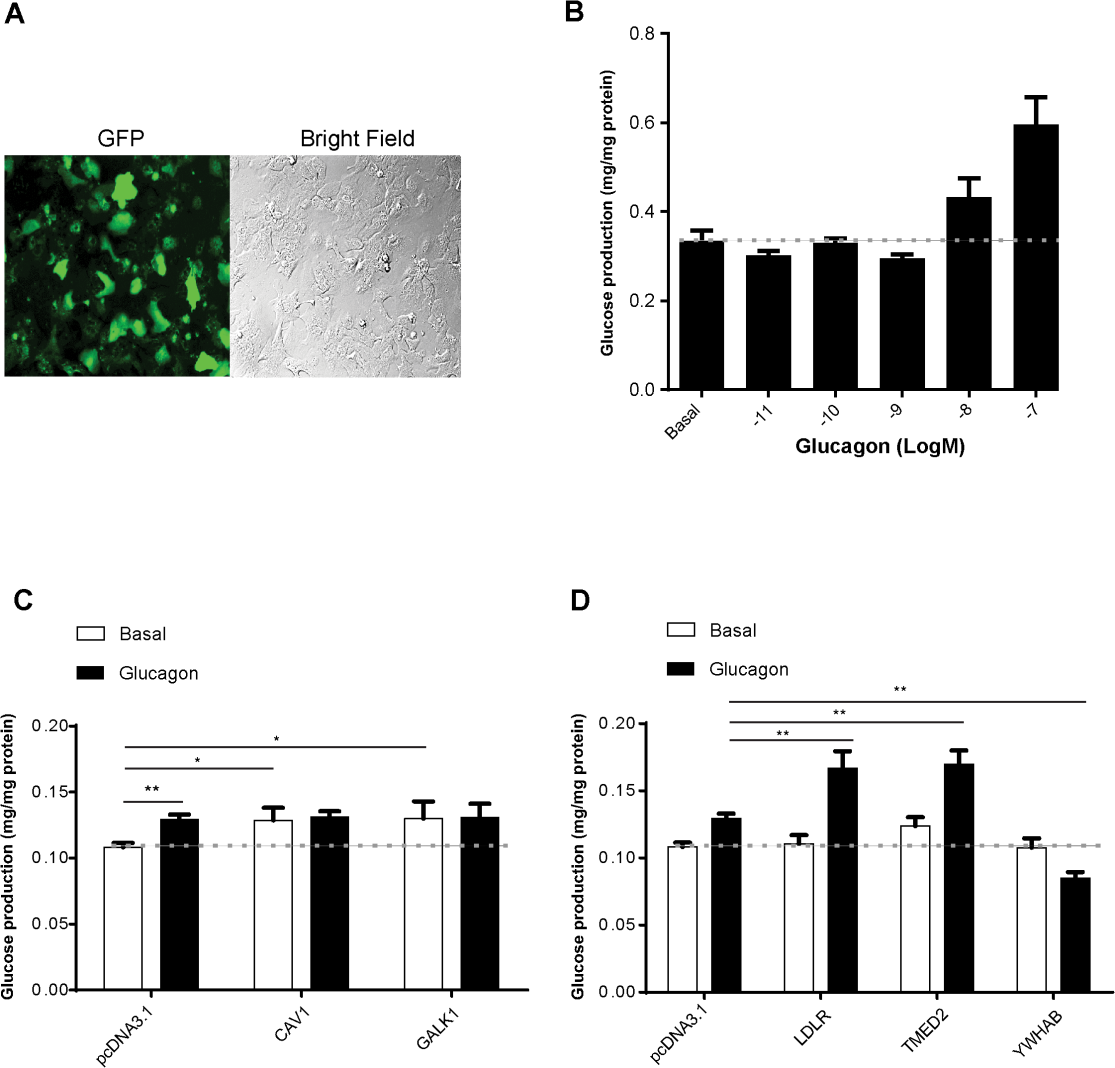
Effects of overexpression of selected GCGR interactors on glucose production in primary mouse hepatocytes. A) Transfection of GFP in primary hepatocytes. B) Dose-response curve of glucagon induced glucose production in primary hepatocytes. 100 nM glucagon treatment significantly increased glucose production in primary hepatocytes (**p<0.01). Readings were normalized to protein amount. Results are presented as mean ± S.E. of three independent experiments. C) Overexpression of CAV1 and GALK1 increased glucose production significantly at basal level (*p< 0.05, **p<0.01 vs cells transfected with pcDNA3.1); D) Overexpression of LDLR and TMED2 increased 100 nM glucagon-stimulated glucose production while YWHAB decreased glucagon-stimulated glucose production significantly (*p< 0.05,**p<0.01 vs cells transfected with pcDNA3.1, N = 3 per group).

GCGRs are expressed in selected tissues in the body but primarily in the liver. Activation of GCGR by glucagon leads to increased glucose production in the liver, achieved by stimulating both glycogenolysis and gluconeogenesis. To evaluate the effect of identified GCGR interactors on GCGR function, we assessed glucose production in primary mouse hepatocytes. A green fluorescent protein (GFP) plasmid was transfected into isolated mouse hepatocytes and the expression efficiency was confirmed to be upwards of 70% (**Fig 4A**). Glucose production in response to glucagon was dose-dependent (**Fig 4B**), indicating proper GCGR signaling in these cells. Five interactors validated by Co-IP and Western blot were transfected into primary hepatocytes. To examine glucose production in these primary hepatocytes, we used the EC_50_ concentration of 100 nM glucagon for treatment. We found that overexpression of CAV1 and GALK1 increased glucose production at the basal (without glucagon treatment) concentration, but not in the presence of glucagon (118.87±9.4%, p<0.05, N = 6 and 120.03±13.0%, p<0.05, N = 6 respectively) (**Fig 4C**). More importantly, overexpression of two interactors (LDLR and TMED2) enhanced glucagon-stimulated glucose production significantly (128.97±12.6%, p<0.01, N = 6 and 131.15±10.3%, p<0.01, N = 6 respectively) but had no effect at the basal level. Overexpression of another interactor, YWHAB, conversely reduced glucagon-induced glucose production significantly (65.59±4.6%, p<0.01, N = 6) (**Fig 4D**) without affecting the basal level glucose production in the primary hepatocytes. The fact that CAV1 and GALK1 only affected glucose production under basal conditions and not in the presence of glucagon, suggested their effects may be independent of GCGR action. For this reason CAV1 and GALK1 were excluded from further functional and mechanistic studies.

### Assessment of cAMP accumulation mediated by selected GCGR Interactors in primary mouse hepatocytes, CHO and HepG2 Cells

**Fig 5:**
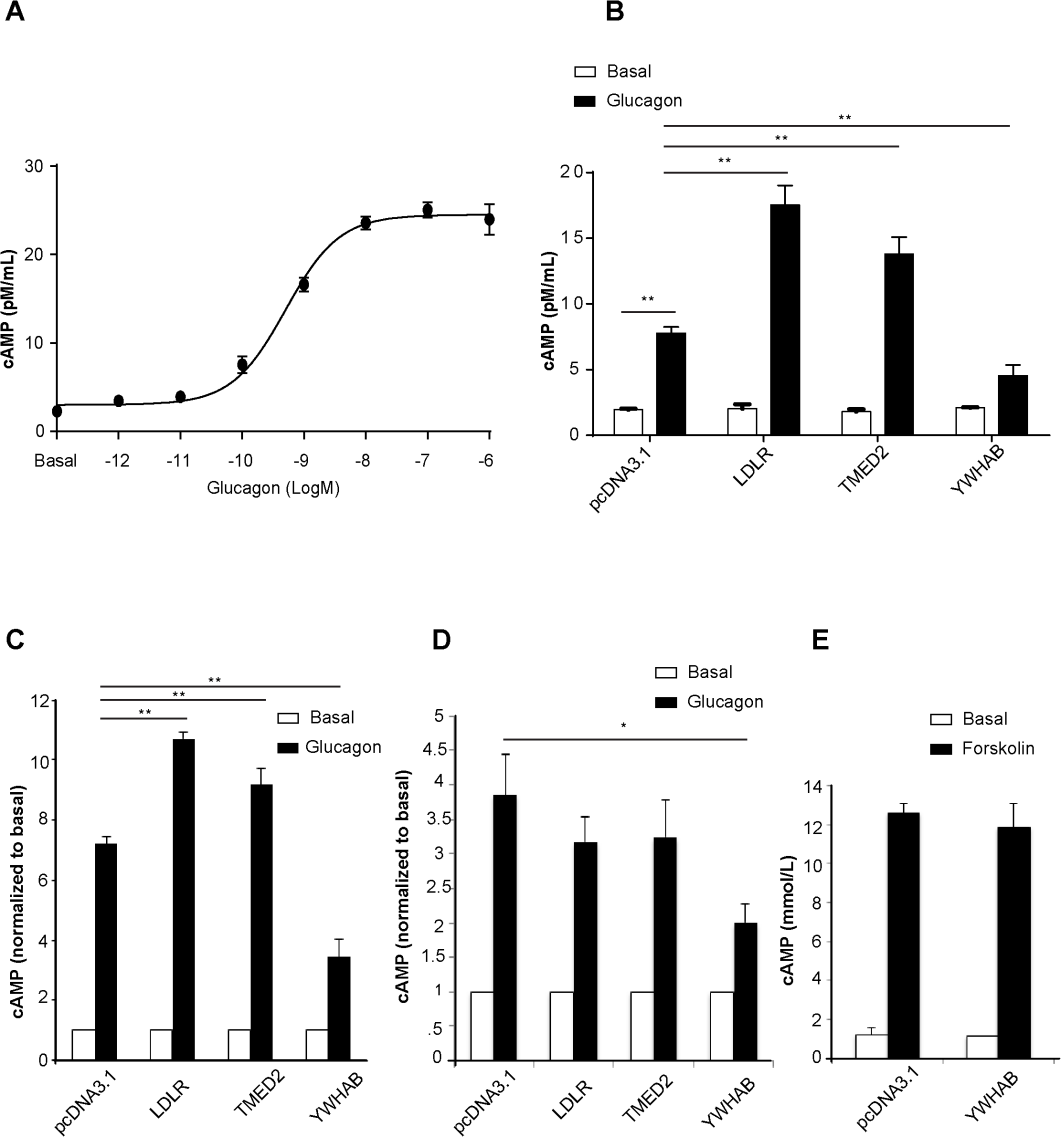
Effects of GCGR interactor overexpression on glucagon-induced cAMP accumulation in primary mouse hepatocytes, CHO cells expressing GCGR and HepG2 stably expressing GCGR. A) Glucagon increased cAMP accumulation in primary mouse hepatocytes, CHO and HepG2-GCGR cells (N = 3 per group) dose-dependently. Primary hepatocytes were transfected with selected GCGR interactors. B) 1 nM glucagon treatment increased cAMP accumulation significantly (**p<0.01). Primary hepatocytes expressing LDLR and TMED2 increased glucagon induced cAMP response significantly (**p<0.01 when compared to hepatocytes transfected with pcDNA3.1). Overexpression of YWHAB in hepatocytes decreased glucagon induced cAMP accumulation (**p<0.01 when compared to hepatocytes transfected with pcDNA3.1).The results are shown as mean ± S.E. Each sample was analyzed in triplicate. C) Overexpression of YWHAB significantly decreased glucagon-induced cAMP accumulation in CHO cells expressing GCGR (*p<0.05 when compared to the pcDNA3.1 control group, N = 3); Overexpression of LDLR and TMED2 significantly increased glucagon-induced cAMP accumulation in CHO cells expressing GCGR (*p<0.05, N = 3), 0.1 nM glucagon. D) Overexpression of LDLR, TMED2 had no significant effect on glucagon-induced cAMP accumulation in HepG2-GCGR cells (p>0.05, N = 4). YWHAB overexpression decreased glucagon-induced cAMP accumulation (*p<0.05, N = 3), 0.1 nM glucagon. E) Overexpression of YWHAB did not affect forskolin-induced cAMP accumulation in CHO cells expressing GCGR.

Upon glucagon binding, GCGR is activated to primarily trigger the cAMP-PKA pathway in which increased cAMP accumulation activates PKA to ultimately elevate glucose production. To determine whether GCGR interactors regulate the gluconeogenic process through the cAMP pathway, we assessed the effect of overexpression of GCGR interactors on cAMP accumulation in response to 0.1 nM glucagon treatment in primary hepatocytes (EC_50_ concentration of cAMP accumulation in response to glucagon, **Fig 5A**). As we anticipated, overexpression of LDLR and TMED2 significantly increased glucagon induced cAMP accumulation to127.74±2.1% (p<0.01, N = 6) and to 80.4±2.13% (p<0.05, N = 6) respectively. In contrast, overexpression of YWHAB significantly suppressed glucagon induced cAMP accumulation by 57.24±7.9% (p<0.01, N = 6, **Fig 5B**). These findings are consistent with previous studies demonstrating that increased cAMP accumulation is the key factor of GCGR mediated glucose production [1].

In line with this observation in primary mouse hepatocytes, in both CHO and HepG2 cells expressing the glucagon receptor, YWHAB overexpression significantly decreased 0.1 nM glucagon induced cAMP accumulation in both CHO and HepG2 cells expressing the glucagon receptor (p<0.05, N = 3, **Fig 5C and 5D**). In CHO cells, overexpression of LDLR and TMED2 led to a significant increase in glucagon induced cAMP accumulation compared to control (**Fig 5C**). Interestingly, no significant difference in cAMP accumulation was found in HepG2-GCGR cells overexpressing LDLR and TMED2. Upon adenylate cyclase activation with forskolin stimulation, YWHAB overexpression did not affect cAMP accumulation in CHO cells expressing GCGR (**Fig 5E**), indicating the effect of YWHAB is mediated via molecules upstream of adenlyate cyclase, likely the GCGR, and that it is not a general inhibitor of adenylate cyclase.

### Gene expression of gluconeogenesis related genes in primary mouse hepatocytes after overexpressing selected interactors

**Fig 6:**
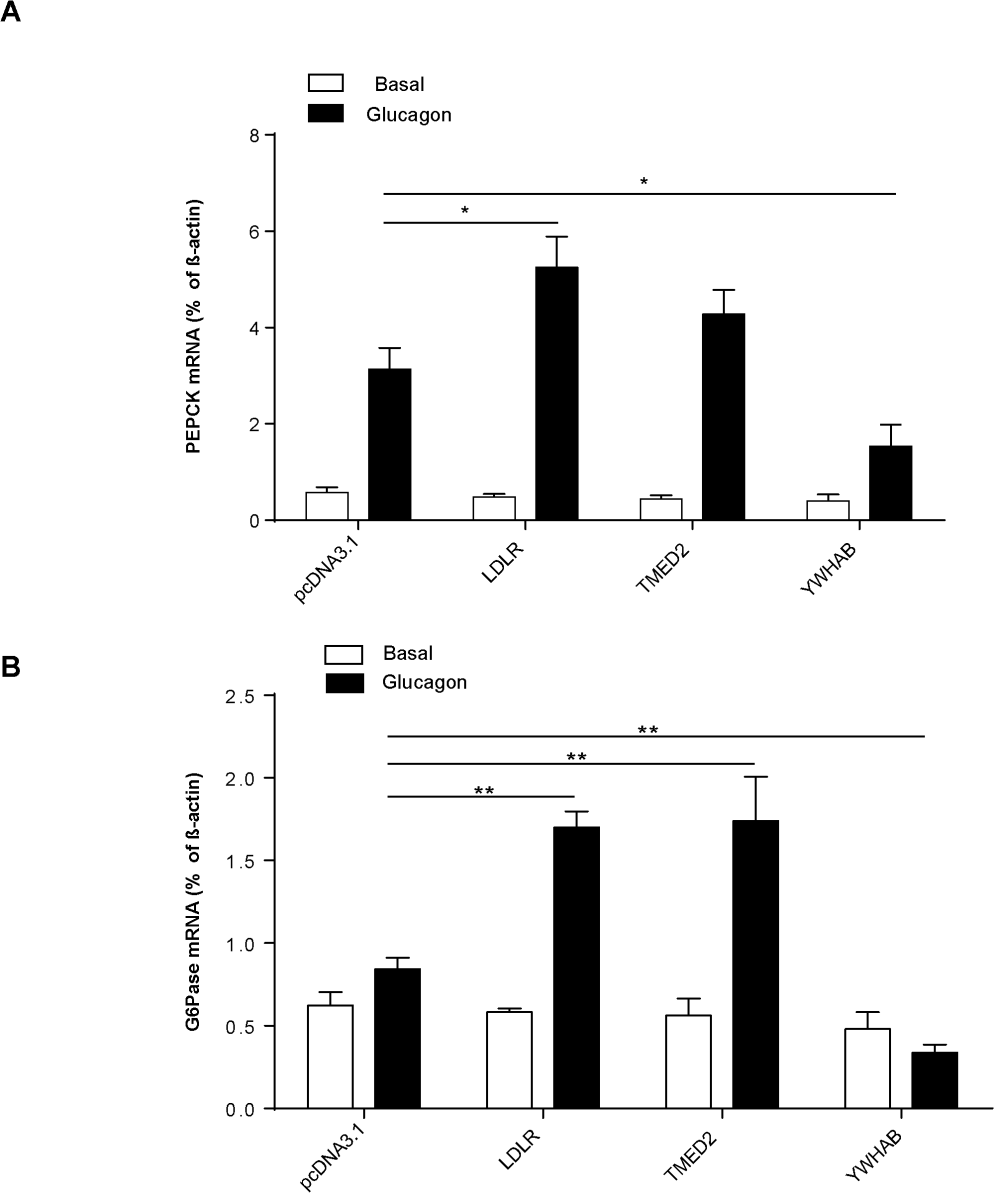
Effects of overexpression of GCGR interactors on expression of gluconeogenesis related genes. Primary hepatocytes were transfected with selected interactors. After pre-incubation of serum-free DMEM, cells were treated with 100 nM glucagon for two hours. mRNA levels of phosphoenolpyruvatecarboxykinase (PEPCK) and glucose-6-phosphatase (G6Pase) were measured by qPCR. A) Overexpression of LDLR increased glucagon-induced PEPCK gene expression in primary mouse hepatocytes (*p<0.05 vs hepatocytes transfected with pcDNA3.1, N = 3 per group). B) Overexpression of LDLR and TMED2 increased glucagon induced G6Pase gene expression in primary mouse hepatocytes (**p<0.01 vs hepatocytes transfected with pcDNA3.1, N = 3 per group). Data are expressed as means ± S.E, presented relative to β-actin transcript expression level.

Gluconeogenesis is responsible for increased glucose production in the liver. It is up-regulated by increased PKA-cAMP signaling/cAMP accumulation [27]. To further understand the mechanism of action of the 3 interactor’s (LDLR, TMED2 and YWHAB) effects on glucose production and GCGR signaling, we examined whether the expression of key gluconeogenic genes were regulated in accordance with altered cAMP accumulation in primary hepatocytes. Phosphoenolpyruvatecarboxykinase (PEPCK) and glucose-6-phosphatase (G6Pase) are critical in gluconeogenesis and GCGR mediated glucose production [28]. PEPCK catalyzes the GTP-dependent conversion of oxaloacetate to phosphoenolpyruvate and G6Pase catalyzes the Mg^2+^-dependent hydrolysis of glucose-6-phosphate to glucose and inorganic phosphate. In our current study, overexpression of LDLR, TMED2 or YWHAB had no effect on gene expression of these two enzymes at the basal level (**Fig 6A and 6B**). More importantly, glucagon induced expression of PEPCK and G6Pase in LDLR overexpressing hepatocytes was significantly increased to 76.52±2.3% and 108±1.7% respectively (p<0.01, N = 6) compared to the control cells (**Fig 6A and 6B**). Similar to LDLR, TMED2 upregulated G6Pase gene expression significantly to 114.15±3.5% (p<0.01, N = 6) in response to glucagon (**Fig 6B**). These findings suggested that LDLR and TMED2 may be involved in up-regulation of gluconeogenesis in the liver. Conversely, YWHAB overexpression significantly reduced glucagon-induced PEPCK and G6Pase gene expression to 52.31±1.4% (p<0.05, N = 6) and 41.61±0.8% (p<0.01, N = 6) respectively (**Fig 6A and 6B**).

### YWHAB decreases cAMP production upon receptor activation prior to adenylate cyclase activation

**Fig 7:**
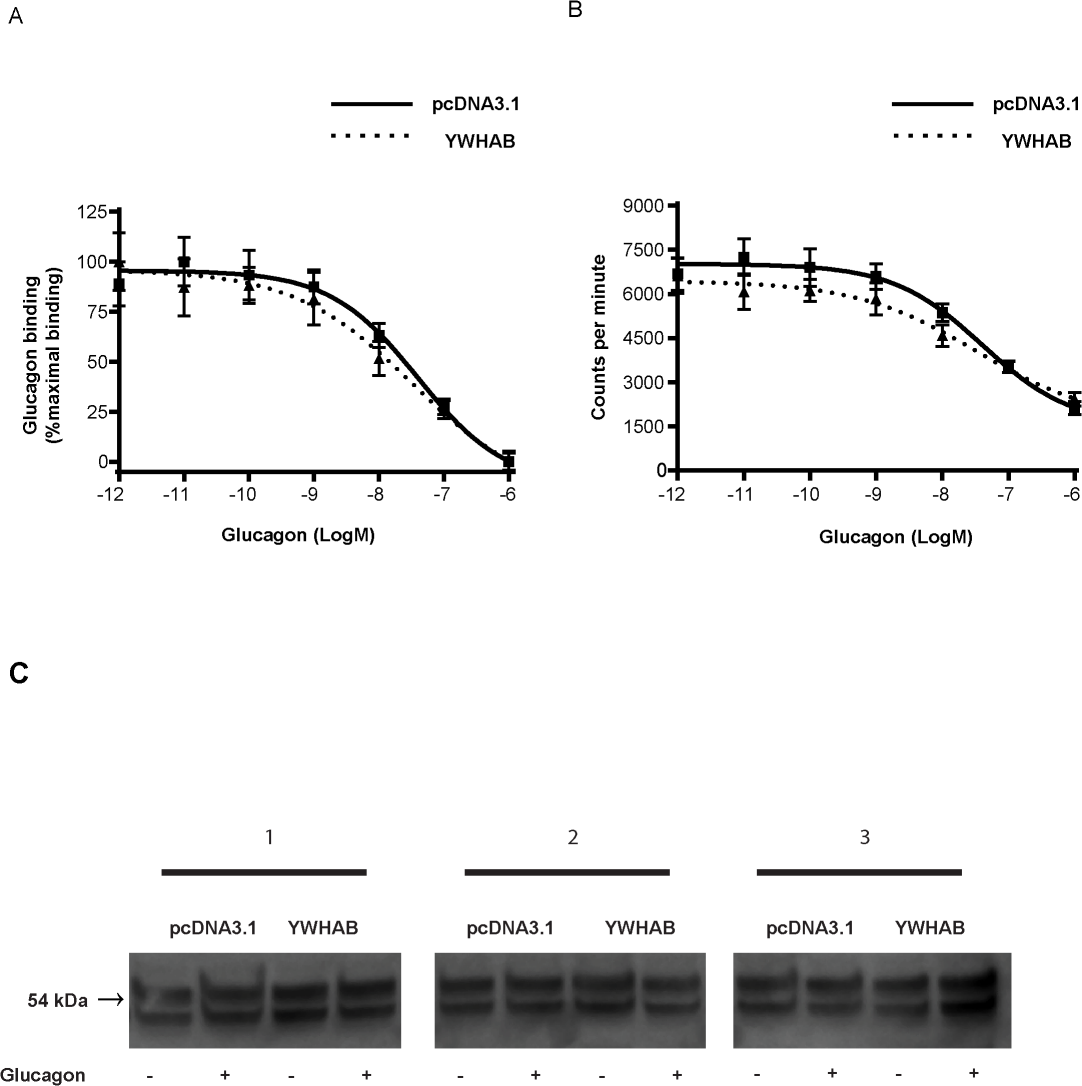
Assessment of the mechanism through which YWHAB decreases cAMP production. A) The affinity and B) radioactive counts of glucagon/GCGR binding in CHO cells transfected with YWHAB (N = 3 per group). C) YWHAB overexpression did not change levels of total serine phosphorylation in HepG2-GCGR cells.

Because YWHAB was the interactor shown to decrease cAMP and glucose production, which is of particular relevance to the treatment of T2D, we sought to further elucidate the mechanism through which YWHAB achieved this observed attenuation. Based on the previously described roles of YWHAB as an adaptor protein, we hypothesized that it may delay recycling of GCGRs that have been phosphorylated and endocytosized from the cell membrane by blocking action of phosphatases [29]. We used a binding assay to examine if YWHAB would change GCGR binding affinity as well as the GCGR total binding (expression) on the cell membrane. The IC_50_ in control cells did not differ from that of YWHAB overexpressing cells. Therefore, YWHAB did not significantly change the binding affinity for glucagon (**Fig 7A**). It is noteworthy that the initial radioactive counts in the binding assay did not differ between these two groups strongly suggesting that the amount of GCGR on the cell membrane was not markedly changed (**Fig 7B)**.

On another aspect, we did not observe any change in serine phosphorylation in HepG2-GCGR cells overexpressing YWHAB compared to control cells suggesting that YWHAB does not alter total serine phosphorylation upon glucagon binding. (**Fig 7C**).

## Discussion

Recently the crystal structure of GCGR, in particular its binding motif with glucagon, was revealed [30] providing new insights into glucagon’s interaction with its receptor. However, GCGR signaling is complex and the factors modulating its activity are not fully understood. In the present study, by using AP-MS we revealed a set of novel interactor proteins of the human GCGR. Three novel interactors (LDLR, TMED2 and YWHAB) were shown to regulate glucagon stimulated glucose production in primary mouse hepatocytes.

Protein-protein interactions (PPIs) have been intensively explored to understand fine tuning of receptor function and signaling in the cells. In recent years AP-MS is emerging as a useful tool for studying PPIs and has been applied to cancer cell signaling and metabolic disease [17, 21,31–32]. More importantly, it allows for the study of activated receptors in a mammalian setting, complementary to our previous study of GLP-1R using the advanced membrane yeast-2-hybrid system, MYTH [21]. Compared to MYTH, AP-MS targets the receptor interactor complex including indirect interactions rather than direct protein-receptor interactions. These features might provide a more comprehensive perspective of the receptor’s interactome in comparison to other discovery tools. As a result of our study, we identified 33 interactors widely involved in cell signal transduction, and metabolism and transport, including two G protein subunits (GNIA2 and GNB2) that are well known to bind to GCGR. Functional enrichment analysis showed most of these GCGR interactors are related to vesicle mediated transport and protein localization, which may be important for GCGR trafficking. Of the 8 interactors (LDLR, CAV1, YWHAQ, YWHAB, YWHAE, TMED2 and TMED10, GALK) selected for validation by Co-IP and Western blot, 3 were not detected in the eluate of GCGR-Flag affinity gel. This could be explained by the possibility that the interaction was too weak to be detected, or that the interaction was indirect. In our earlier study using a similar AP-MS strategy, we identified a set of GLP-1R interactors [17]. The native ligands of GLP-1R (GLP-1) and GCGR (glucagon) are transcribed from the same gene and spliced from the same propeptide, proglucagon. Since GLP-1R and GCGR are structurally related and belong to class B GPCR family, it is not a surprising that the two receptors shared similar functional clusters of interacting proteins including G proteins (i.e. GNAI2), cell signaling (i.e. YWHAQ and PGRMC1), and molecular transport (i.e. CAV1 and TMED10). However, compared to GLP-1R, GCGR had a unique functional group of interactors involved in lipid and carbohydrate metabolism (LDLR and GALK1), in accordance with its unique role in regulating glucose homeostasis.

Further study of glucose production in primary mouse hepatocytes supported our previous hypothesis that these cells possess interactors that regulate activated GCGR. Over expression of 3 GCGR interactors (LDLR, TMED2 and YWHAB) had significant effects on glucagon stimulated glucose production (GSGP). Interestingly, overexpression of two liganded interactors CAV1 (an interactor of both liganded and unliganded GCGR) and GALK enhanced basal glucose production but had no effect on glucagon-stimulated glucose production in primary mouse hepatocytes. It is possible that these two interactors require involvement of other factors to regulate GCGR. On the other hand, we also found that unliganded GCGR interactor LDLR had a significant effect on glucagon-stimulated glucose production. This could be due to its profound and complex involvement in lipid metabolism, which also links with glucose production. The reported major role of LDLR is to bind and internalize circulating cholesterol-containing lipoprotein particles which is required for LDL catabolism [33]. Although increased hepatic VLDL is a characteristic of T2D [34], LDL levels remains normal. This is possibly due to increased LDL catabolism in early T2D patients who are hyperinsulinemic [35]. Further studies also suggested possible links between LDLR up-regulation and insulin signaling [36, 37]. Therefore in our experimental paradigm, although LDLR was identified as an interactor of unliganded GCGR, elevated LDLR level may increase lipid metabolism which could in turn affect glucose production. Interestingly, beta-arrestin 2 binds to LDLR and modulates LDLR endocytosis [38], and beta-arrestin 1 can interact with class B GPCR, for example GLP-1R and gastric inhibitor peptide receptor (GIPR),to mediate their effects on Gs-cAMP signaling [12, 39]. Therefore, in our model, LDLR may interact with GCGR and regulate glucagon induced cAMP signaling through beta-arrestin 1 in primary hepatocytes.

The interactor TMED2 is reported to be part of the coat protein complex (coatomer) [40] and is important to vesicle transport by interacting with ADP-ribosylation factor 1 (ARF1) [41–43]. Recently it was shown to play a role in trafficking of GPCRs, including protease-activated receptor-2 (PAR-2) and calcium sensing receptor (CaSR), from the ER/Golgi to the plasma membrane [44]. These studies showed that TMED2 could decrease receptor degradation and stabilize the receptor in the ER, possibly enhancing plasma membrane targeting. In particular, similar to our finding, TMED2 was identified in the yeast-two-hybrid screens with CaSR C-terminus [44], suggesting it is an important partner of GPCRs. In the context of this study, elevated levels of TMED2 upon glucagon stimulation might increase the trafficking of GCGR from Golgi to plasma membrane thereby enhancing the glucagon/GCGR pathway. This assumption is supported by increased cAMP accumulation and elevated expression of downstream gluconeogenesis related genes, supporting a positive role for TMED2 in the regulation of GCGR activity in glucose production in the liver.

The interactor 14-3-3 protein beta, also known as tyrosine 3-monooxygenase/tryptophan 5-monooxygenase activation protein, beta (YWHAB) belongs to the 14-3-3 protein family. By bioinformatic analysis in a T2D mouse liver membrane proteome study, YWHAB was revealed as a novel protein with correlations to a set of membrane proteins that were differentially expressed in T2D [45]. YWHAB regulates the activity of ChREBP (carbohydrate response element-binding protein), which regulates expression of genes involved in hepatic glycolysis and lipogenesis [46]. Furthermore, 14-3-3 protein zeta was shown to co-immunoprecipitate with GSK3 and tau in the brain [47, 48] and it also facilitates GSK3-catalyzed tau phosphorylation in HEK293 cells [49]. Although the role of GSK3 in glycogen synthesis is yet to be defined in different tissues, it is reasonable to speculate in our scenario that it could be a downstream effector of YWHAB that reduces glucose production by the regulation of glycogen synthesis. Although YWHAB was not reported to affect cAMP signaling, its overexpression caused suppression of gluconeogenesis in mouse primary hepatocytes and decreased cAMP accumulation (**Figs 4D and 5A**).

One possible mechanism through which YWHAB decreases cAMP may be through an interaction between arrestins and GCGR. Upon binding of a ligand to a GPCR, desensitization of the receptor begins immediately to limit the potential harmful effects of continual activation [50]. This desensitization is the result of concerted action of GRKs, arrestins and other protein kinases [50]. Phosphorylation of a stimulated receptor by GRKs leads to recruitment of arrestins to physically uncouple the receptor from its associated G protein. Additionally, protein kinases, such as PKA, also directly phosphorylate the receptor [50], as well as GRKs to increase their activity [50]. Since YWHAB was shown to decrease cAMP production upon glucagon stimulation without acting as a general inhibitor of cAMP production, and did not lead to any decreases in ligand affinity nor in cell surface expression of GCGR, we predict that YWHAB may play a role in mediating the interaction between arrestins and the GCGR. We did not observe any change in glucagon-stimulated total serine phosphorylation in cells overexpressing YWHAB, suggesting that GCGR phosphorylation is not regulated by YWHAB to affect activity. However, further examination of the specific serine phosphorylation status of GCGR is required to rule this out completely. Another potential mechanism through which YWHAB may lead to a decrease in cAMP production is through a similar interaction between PKA and the receptor, or a complex involving PKA, GRKs and the receptor.

## Conclusions

In mammalian cells, using the full-length human GCGR as a probe, we revealed a complex GCGR protein interactome. Selected GCGR interactors were shown to regulate glucagon-stimulated glucose production by modulating glucagon-induced cAMP accumulation and gluconeogenesis. This correlated with specific changes in key gluconeogenic genes in mouse hepatocytes. One interactor of interest, YWHAB, may be involved in GCGR desensitization, attenuating glucagon-stimulated glucose production. These findings highlighted the feasibility of employing the AP-MS/MS strategy to identify novel GPCR interactors. This study provides further novel insight into GCGR signaling and identifies novel targets for the development of anti-glucagon action agents.

## Supporting Information

S1 Table Primer sequences for qPCR. (DOCX)

S2 Table GCGR interactors identified from the AP/MS screen. (DOCX)
