# Supplementary material for: The Identification of Novel Protein-Protein Interactions in Liver that Affect Glucagon Receptor Activity
Source: PLoS One. 2015 Jun 15;10(6):e0129226. doi: 10.1371/journal.pone.0129226 (PMC4468146; doi:10.1371/journal.pone.0129226)
Supplement: S1 Table — (DOCX) [file pone.0129226.s001.docx]

| Gene | Full Name | Primer Sequence |
| --- | --- | --- |
| G6Pase | Glucose 6-phosphotase | 5’- TCTGTCCCGGATCTACCTTG -3’ Forward  5’- GTAGAATCCAAGCGCGAAAC -3’ Reverse |
| PEPCK | Phosphoenolpyruvate carboxykinase | 5’- GTGAGGAAGTTCGTGGAAGG -3’ Forward  5’- TCTGCTCTTGGGTGATGATG -3’ Reverse |
| ACTB | β-actin | 5’- CTGAATGGCCCAGGTCTGA -3’ Forward  5’- CCCTGGCTGCCTCAACAC -3’ Reverse |

Supplemental Table 1. Primer sequences used for qPCR
