# Supplementary material for: The Identification of Novel Protein-Protein Interactions in Liver that Affect Glucagon Receptor Activity
Source: PLoS One. 2015 Jun 15;10(6):e0129226. doi: 10.1371/journal.pone.0129226 (PMC4468146; doi:10.1371/journal.pone.0129226)
Supplement: S2 Table — (DOCX) [file pone.0129226.s002.docx]

| Supplemental Table 2. Characterization of GCGR interactors identified by AP-MS  **Uniprot** | **Protein name** | **Gene name** | **Biological process** | **Subcelluar location** | **Unliganded GCGR interactors** | **Liganded GCGR interactors** |
| --- | --- | --- | --- | --- | --- | --- |
|  |  |  |  |  |  |  |
| **G3V2K7** | Transmembrane emp24 domain-containing protein 10 | TMED10 | transport | Membrane |  | ✓ |
| **Q15363** | Transmembrane emp24 domain-containing protein 2 | TMED2 | ER-Golgi transport; Protein transport | Cytoplasmic vesicle membrane, ER Membrane, Golgi apparatus |  | ✓ |
| **P06703** | Protein S100-A6 | S100A6 | axonogenesis;signal transduction | Cytoplasm. Cell membrane, Nucleus |  | ✓ |
| **P26447** | Protein S100-A4 | S100A4 | epithelial to mesenchymaltransition;positive regulation of I-kappaB kinase/NF-kappaB cascade | Nucleus, cytoplasm |  | ✓ |
| **Q92542** | Nicastrin | NCSTN | Notch signaling pathway | Membrane |  | ✓ |
| **P31153** | S-adenosylmethionine synthase isoform type-2 | MAT2A | One-carbon metabolism | Cytoplasm |  | ✓ |
| **P54709** | Sodium/potassium-transporting ATPase subunit beta-3 | ATP1B3 | Ion transport;Sodium/potassium transport | Cell membrane, caveola |  | ✓ |
| **Q9UBB4** | Ataxin-10 | ATXN10 | celldeath;neuron projection development | Cytoplasm |  | ✓ |
| **P31946** | 14-3-3 protein beta/alpha | YWHAB | MAPK cascade;Ras protein signal transduction | Cytoplasm, nucleus |  | ✓ |
| **Q96BI3** | Gamma-secretase subunit APH-1A | APH1A | Notch signaling pathway | Endoplasmic reticulum membrane, Golgi apparutus |  | ✓ |
| **P62879** | Guanine nucleotide-binding protein G(I)/G(S)/G(T) subunit beta-2 | GNB2 | G-protein coupled receptor signaling pathway | Cytoplasm | ✓ |  |
| **Q9BZG1** | Ras-related protein Rab-34 | RAB34 | Protein transport | Cytoplasm. Golgi apparatus | ✓ |  |
| **P01130** | Low-density lipoprotein receptor | LDLR | Cholesterol metabolism;Endocytosis;Host-virus interaction | Cell membrane | ✓ |  |
| **P02786** | Transferrin receptor 1 | TFR1 | Endocytosis;Host-virus interaction | Cell membrane | ✓ | ✓ |
| **P62258** | 14-3-3 protein epsilon | YWHAE | Host-virus interaction | Cytoplasm | ✓ | ✓ |
| **P21796** | Voltage-dependent anion-selective channel protein 1 | VDAC1 | Apoptosis;Host-virusinteraction;Ion transport | Mitochondrion outer membrane, Cell membrane | ✓ | ✓ |
| **P51570** | Galactokinase | GALK1 | Carbohydrate metabolism;Galactose metabolism | Cytoplasm | ✓ | ✓ |
| **P27348** | 14-3-3 protein theta | YWHAQ | cellular membrane organization;intrinsic apoptotic signaling pathway | Cytoplasm | ✓ | ✓ |
| **P10253** | Lysosomal alpha-glucosidase | GAA | cardiac muscle contraction;glucose metabolic process | Lysosome membrane | ✓ | ✓ |
| **P84077** | ADP-ribosylation factor 1 | ARF1 | ER-Golgi transport;Protein transport | Golgi, cytoplasm | ✓ | ✓ |
| **P30520** | Adenylosuccinatesynthetaseisozyme 2 | ADSS | Purine biosynthesis | Cytoplasm | ✓ | ✓ |
| **Q03135** | Caveolin-1 | CAV1 | Endocytosis | Plasma membrane | ✓ | ✓ |
| **P04899** | Guanine nucleotide-binding protein G(i), alpha-2 subunit | GNAI2 | Cell cycle;Cell division | Plasma membrane | ✓ | ✓ |
| **P50281** | Matrix metalloproteinase-14 precursor | MMP14 | angiogenesis;astrocyte cell migration | Plasma membrane | ✓ | ✓ |
| **O00264** | Membrane associated progesterone receptor component 1 | PGRMC1 | axon guidance | Plasma membrane | ✓ | ✓ |
| **Q92597** | NDRG1 protein | NDRG1 | DNA damage response, signal transduction by p53 class mediator;cellular response to hypoxia | Plasma membrane, cytoplasm | ✓ | ✓ |
| **Q58F24** | Plasma membrane calcium-transporting ATPase 1 | ATP2B1 | ATP binding | Plasma membrane | ✓ | ✓ |
| **Q9NP72** | Ras-related protein Rab-18 | RAB18 | Protein transport | Plasma membrane | ✓ | ✓ |
| **P62491** | Ras-related protein Rab11 | RAB11 | Cell cycle;Protein transport | Plasma membrane | ✓ | ✓ |
| **P05023** | Sodium/potassium-transporting ATPase alpha-1 chain precursor | ATP1A1 | Ion transport;Sodium/potassium transport | Plasma membrane | ✓ | ✓ |
| **P16615** | Sarcoplasmic/endoplasmic reticulum calcium ATPase 2 | ATP2A2 | Calcium transport;Ion transport | ER membrane | ✓ | ✓ |
| **O15270** | Serine palmitoyltransferase 2 | SPTLC2 | Lipid metabolism;Sphingolipid metabolism | ER membrane | ✓ | ✓ |
| **O95292** | Vesicle-associated membrane protein-associated protein B | VAPB | Host-virus interaction;Unfolded protein response | ER membrane | ✓ | ✓ |
